# Estimated Cardiorespiratory Fitness Attenuates the Impacts of Sarcopenia and Obesity on Non-Alcoholic Fatty Liver in Korean Adults

**DOI:** 10.3390/ijerph17113902

**Published:** 2020-05-31

**Authors:** Inhwan Lee, Jeonghyeon Kim, Hyunsik Kang

**Affiliations:** College of Sport Science, Sungkyunkwan University, Suwon 16419, Korea; ansh00@naver.com (I.L.); zzagkim115@naver.com (J.K.)

**Keywords:** fatty liver disease, physical fitness, physical activity, sarcopenia, obesity

## Abstract

This population-based, cross-sectional study examined the preventive role of non-exercise-based estimation of cardiorespiratory fitness (eCRF) against the impacts of sarcopenia and obesity on the non-alcoholic fatty liver (NAFL) in Korean adults. Data were obtained from the 2008–2011 Korea National Health and Nutrition Examination Surveys IV and V (*n* = 14,015 Koreans aged ≥ 18 years, 64% women). eCRF was calculated with the age- and sex-specific algorithms, and classified as lower (lowest 25%), middle (middle 50%) and upper (highest 25%). Individuals were classified as optimal (i.e., the absence of both sarcopenia and obesity), sarcopenia (i.e., the presence of sarcopenia), obesity (i.e., the presence of obesity) or sarcopenic obesity (i.e., the coexistence of sarcopenia and obesity). Limited to the sarcopenia phenotype, the adjusted odds ratio (OR) of NAFL was 2.2 (95% confidence interval, CI, 1.5–3.1) for the lower eCRF, 1.6 (95% CI, 1.3–2.1) for the middle eCRF and 2.1 (95% CI, 1.4–3.1) for the upper eCRF, compared to the optimal phenotype. Limited to the obesity phenotype, the adjusted OR of NAFL was 2.9 (95% CI, 2.0–4.2) for the lower eCRF, 3.5 (95% CI, 2.7–4.6) for the middle eCRF and 1.8 (95% CI, 1.2–2.8) for the upper eCRF, compared to the optimal phenotype. Limited to the sarcopenic obesity phenotype, the adjusted OR of NAFL was 5.9 (95% CI, 4.3–8.2) for the lower eCRF, 4.2 (95% CI, 3.2–5.5) for the middle eCRF and 2.5 (95% CI, 1.5–4.1) for the upper eCRF, compared to the optimal phenotype. The current findings suggest that high eCRF attenuates the individual and synergistic impacts of sarcopenia and obesity on NAFL in Korean adults.

## 1. Introduction

Non-alcoholic fatty liver disease (NAFLD) encompasses two subtypes of non-alcoholic fatty liver (NAFL) and non-alcoholic steatohepatitis (NASH). The global prevalence of NAFLD is estimated to be approximately 25% in the general population [1], although it varies from 15% to 70% depending on age, sex, ethnicity and diagnostic method [2,3]. The prevalence of NAFLD is projected to rise, due to an increase in Westernized affluent and sedentary lifestyles [4]. NAFL represents an excessive accumulation of hepatic fat in the absence of any significant inflammation or fibrosis. Pathologically, NAFL is closely associated with metabolic complications, such as hyperinsulinemia or dyslipidemia, resulting in such adverse health outcomes as type 2 diabetes [5] and cardiovascular diseases (CVDs) [6].

Obesity and cardiorespiratory fitness (CRF), which reflects the ability of the respiratory and circulatory systems to deliver oxygen to working skeletal muscles during exercise, have been well established as independent predictors of metabolic syndrome [7], diabetes [8] and CVDs [9]. The two lifestyle risk factors are also associated with NAFL [10,11]. In addition, sarcopenia is known as another novel risk factor of NAFL in Western [12,13] and Asian populations [14,15,16], collectively suggesting that an interaction among adipose tissue, skeletal muscle and the cardiorespiratory system may play an important role in the pathophysiology of NAFL [17]. In Korea, the prevalence of NAFL is steadily rising in those middle aged and older, particularly in men with central obesity [18], and in individuals with sarcopenia [16] or poor CRF [19]. Poor CRF increases the risk of both sarcopenia and visceral obesity in apparently healthy Korean adults [19]. Therefore, examining the individual and synergistic impacts of lifestyle risk factors on NAFL will provide critical information and help develop better therapeutic strategies for patients with NAFL.

An objective measurement of CRF, which requires laboratory-based assessments with gas analysis, is often limited due to such requirements as specialized equipment and trained personnel, as well as being time-consuming, especially in a large population-based study. Fortunately, CRF can be estimated with sex- and age-specific algorithms, developed on the basis of routinely-obtained health indictors, with acceptable accuracy [20]. To the best of our knowledge, however, no previous study has investigated whether or not eCRF is a moderator in determining the association of sarcopenia and/or obesity with NAFL in the Korean population. This study aimed to investigate the protective role of eCRF against the synergistic impacts of sarcopenia and obesity on NAFL in Korean adults.

## 2. Materials and Methods 

### 2.1. Data Source and Study Participants

The data used in this study were obtained from the Korea National Health and Nutrition Examination Surveys (KNHANES) IV and V, conducted in the Republic of Korea from 2007 to 2011 [20]. In brief, KNHANES is a continuous survey of South Korea. Each survey consists of independent sets of non-institutionalized civilians aged 1 year and older from the South Korean population. All participants were randomly assigned from 600 randomly selected districts of cities and provinces in South Korea. KNHANES IV comprises survey 1 in 2007 (*n* = 4594), survey 2 in 2008 (*n* = 9744) and survey 3 in 2009 (*n* = 10,533). KNHANES V comprises survey 1 in 2010 (*n* = 8958) and survey 2 in 2011 (*n* = 8518). All statistics of this survey have been calculated using sample weights assigned to sample participants. A detailed description of the sample weights is available elsewhere [21]. 

**Figure 1:**
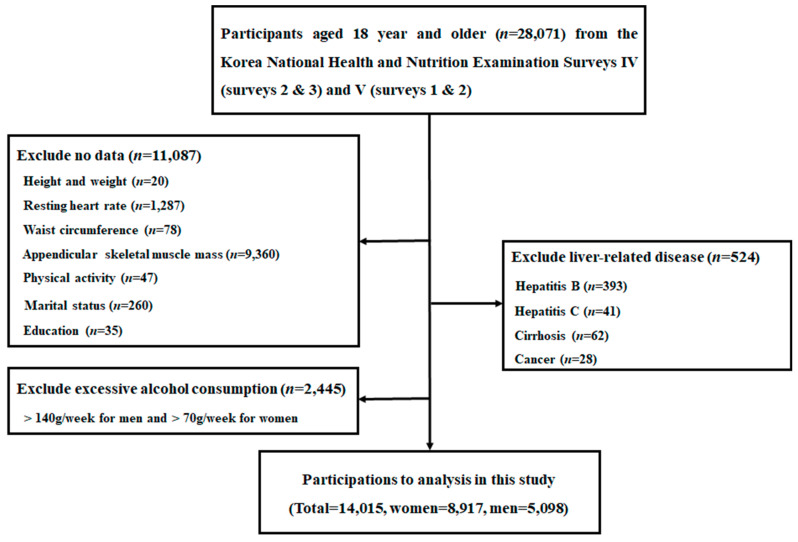
Flow chart of eligible participants in the study.

For the current study, we initially selected 28,071 adults aged 18 years and older from KNHANES IV (survey 2 and survey 3) and V (survey 1 and survey 2). We then excluded individuals who had no data available regarding height and weight (*n* = 20), resting heart rate (*n* = 1287), waist circumference (WC) (*n* = 78), appendicular skeletal muscle mass (*n* = 9360), physical activity (*n* = 47), marital status (*n* = 260) or education (*n* = 35) at baseline. Individuals who met the following criteria were additionally excluded: (1) alcohol abuse (*n* = 2445), defined as a weekly alcohol consumption > 140 g/week for men and > 70 g/week for women; 2) positive serologic markers for hepatitis B (*n* = 393) or hepatitis C virus (*n* = 41); and (3) the presence of liver cirrhosis (*n* = 62) or liver cancer (*n* = 28). Consequently, a total of 14,015 adults (8917 women) were included in the final data analyses (Figure 1). The Institutional Review Board of the Korea Centre for Disease Control and Prevention in accordance with the Declaration of Helsinki reviewed and approved the KNHANES (Nos. 2008-04EXP-01-C, 2009-01CON-03-2C, 2010-02CON-21-C, 2011-02CON-06C).

### 2.2. Study Variables

#### 2.2.1. Definition of Obesity and Sarcopenia

Asians, including Koreans, have a higher risk of central obesity than Western populations, due to a higher chance of central fat deposition, despite having lower BMI [22]. More importantly, BMI fails to account for changes in body composition with aging, specifically the age-related decline in muscle mass, strength and function [23]. Consequently, WC is known as a better indicator of visceral adiposity, and is more closely associated with insulin resistance (IR) and its related metabolic disorders such as NALFD [24,25], especially in Koreans [26,27]. In this study, therefore, the same Korean-specific WC threshold for metabolic syndrome was used to determine central obesity: WC ≥ 90 cm in men or ≥85 cm in women [28]. 

Appendicular skeletal muscle mass (ASM) was defined as the sum of the muscle mass in the arms and legs, measured with dual energy X-ray absorptiometry (DEXA) (DISCOVERY-W fan-beam densitometer, Hologic, Inc., Bedford, MA, USA). Sarcopenic index was calculated with ASM (kg) divided by body weight (kg), and the index values that were < 1 standard deviation below the mean of the sex-specific healthy reference group (i.e., aged 20–39 years) from the same KNHANES IV and V database were considered sarcopenic [29].

Based on the sarcopenia and obesity assessments, individuals were classified as optimal (i.e., the absence of body sarcopenia and obesity), sarcopenia (i.e., the presence of sarcopenia), obesity (i.e., the presence of obesity) or sarcopenic obesity (i.e., the coexistence of sarcopenia and obesity). 

#### 2.2.2. Calculation of eCRF

eCRF was calculated in units of metabolic equivalents (METs) [20]; eCRF (METs) = 2.77 (sex) − 0.10 (age) − 0.17 (body mass index) − 0.03 (resting heart rate) + 1.00 (physical activity score) + 18.07. Physical activity score was assessed with the Johnson Space Centre Physical Activity Rating scale, which was developed to provide a self-rating in the range of 0 (avoid physical activities whenever possible) to 7 (heavy physical activities done regularly for more than 3 hours per week) [30]. eCRF was then classified into lower (lowest 25%), middle (middle 50%) and upper (highest 25%), on the basis of sex- and age-specific tertiles of individual distributions.

#### 2.2.3. Definition of Hepatic Steatosis

Hepatic steatosis was determined using the following two indices of suspected hepatic steatosis.

NAFLD liver fat score (NAFLD-LFS) [31]

NAFLD-LFS = −2.89 + 1.18 (metabolic syndrome; yes = 1, no = 0) + 0.45 (type 2 diabetes; yes = 2, no = 0) + 0.15 (insulin) + (0.04 × alanine aminotransferase) − 0.94 × (aspartate aminotransferase/alanine aminotransferase), with fasting serum concentrations of insulin (µU/ml), alanine aminotransferase (U/L) and aspartate aminotransferase (U/L). Metabolic syndrome (Mets) was determined according to a revised National Cholesterol Education Program definition [32] with the adoption of a Korean-specific WC threshold [28]. 

Hepatic steatosis index (HSI) [33].

HSI = 8 (alanine aminotransferase/aspartate aminotransferase ratio) + body mass index (+ 2 if diabetes, + 2 if female), with the cut-off point of 30 for steatosis was used.

#### 2.2.4. Clinical and Laboratory Measurements 

Participants were required to complete self-administered questionnaires regarding smoking, alcohol habits, physical activity, income, marital status, education and past medical history. Height, weight, WC, systolic blood pressure (SBP), diastolic blood pressure (DBP) and resting heart rate (RHR) were assessed with standardized procedures by trained persons. Body mass index (BMI) was calculated as weight divided by height (kg/m^2^). 

Fasting venous blood sampling was performed after overnight fasting to determine concentrations of glucose, total cholesterol (TC), triglycerides (TG), high-density lipoprotein cholesterol (HDLC), insulin, alanine aminotransferase (ALT), aspartate aminotransferase (AST) and platelet. Homeostasis model assessment of insulin resistance (HOMA-IR) was calculated; HOMA-IR = fasting insulin (uU/L) × fasting glucose (nmol/L)/22.5. A detailed description of the clinical and laboratory measurements is available elsewhere [22].

### 2.3. Statistical Analyses

All variables were checked for normality, both visually and through the Kolmogorov–Smirnov test, and subjected to an appropriate transformation, if necessary, prior to statistical analyses. Descriptive statistics were presented as means and standard deviations for continuous variables, and as percentages for categorical variables. Parametric and non-parametric statistics were also used to compare continuous and categorical variables, respectively, among sarcopenia and obesity-based phenotypes (i.e., optimal, sarcopenia, obesity and sarcopenic obesity) stratified by eCRF categories (i.e., lower, middle and upper). Odd ratios (ORs) and 95% confidence intervals (95% CIs) of sarcopenia and obesity-based phenotypes for NAFL were calculated in each eCRF category using multiple logistic regression analyses before and after adjustments for all measured covariates. Alpha was set at 0.05. All statistical analyses were performed using the SPSS-PC statistical software (version 23.0, SPSS Inc., Chicago, IL, USA).

## 3. Results

Table 1 represents the descriptive statistics of study participants. In general, men had more favorable profiles of marital status and education, and higher rates of physical inactivity, smoking, heavy alcohol consumption, hypertension, diabetes and metabolic syndrome, in conjunction with a higher rate of glucose-lowering medications and a lower rate of lipid-lowering medications than women. Men had poorer blood chemistry profiles than women, as shown in FBG, TG, AST, ALT, NAFLD-LFS, HSI and HDLC. 

Compared to optimal, anthropometrics (i.e., age, BMI, percent body fat, WC and SMI), SES (i.e., marital status and education) and health conditions (i.e., smoking, alcohol consumption, inactivity, hypertension, diabetes, metabolic syndrome, menopause, glucose and lipid-lowering medications, and markers of NAFL) became worse in the order of sarcopenia, obesity and sarcopenic obesity, in the lower, middle and upper eCRF categories. Compared to optimal, blood chemistry profiles (i.e., FBG, insulin, HOMA-IR, TC, HDL-C, TG, AST and ALT) also became worse in the order of sarcopenia, obesity and sarcopenic obesity, in the lower, middle and upper eCRF categories (refer to Tables S1–S3 in the Supplementary Materials). 

Table 2 represents the impacts of sarcopenia and obesity on the prevalence of NAFL, stratified by eCRF levels. Significant inverse, linear trends in the prevalence of NAFL were found according to incremental eCRF categories (from lower to upper) in all the sarcopenia and obesity-based phenotypes.

Table 3 represents the ORs and 95% CIs of sarcopenia and obesity-based phenotypes for NAFL, stratified by eCRF levels. Limited to the sarcopenia phenotype, the adjusted OR of NAFL was 2.2 (95% CI, 1.5–3.1) for the lower eCRF, 1.6 (95% CI, 1.3–2.1) for the middle eCRF and 2.1 (95% CI, 1.4–3.1) for the upper, compared to the optimal phenotype. Limited to the obesity phenotype, the adjusted OR of NAFL was 2.9 (95% CI, 2.0–4.2) for the lower eCRF, 3.5 (95% CI, 2.7–4.6) for the middle eCRF and 1.8 (95% CI, 1.2–2.8) for the upper eCRF, compared to the optimal phenotype. Limited to the sarcopenic obesity phenotype, the adjusted OR of NAFL was 5.9 (95% CI, 4.3–8.2) for the lower eCRF, 4.2 (95% CI, 3.2–5.5) for the middle eCRF and 2.5 (95% CI, 1.5–4.1) for the upper eCRF, compared to the optimal phenotype. Together, the findings of the current study suggest that high eCRF attenuates the individual and synergistic impacts of sarcopenia and obesity on NAFL.

## 4. Discussion

In this population-based study, we examined the impacts of sarcopenia and obesity on NAFL, stratified by eCRF levels, in Korean adults, and found that NAFL was positively associated with sarcopenia and/or obesity, and inversely associated with eCRF. To the best of our knowledge, this is the first study to report that high eCRF attenuates the individual and synergistic impacts of sarcopenia and obesity on NAFL in Korean adults. 

The findings of the current study are in accordance with previous studies reporting obesity and sarcopenia as important risk factors for NAFL. In an average 4.5-year follow-up cohort study, involving 77,425 metabolically healthy adults at baseline, Chang et al. [34] showed that overweight and obese individuals had higher hazard ratios of incident NAFLD (HR = 2.15, 95% CI = 2.06–2.26 and OR = 3.55, 95% CI = 3.37–3.74, respectively) compared with normal-weight individuals. By analyzing the data obtained from 29,994 Korean adults aged 18 years and older who had a routine health examination, Kwon et al. [35] showed that obese individuals had a higher prevalence of NAFLD (50.1% vs. 12.6%) compared to non-obese individuals. Yang et al. [36] examined the association between NAFLD and obesity-related metabolic syndrome in a sample of 614 Korean participants recruited from a local community, and found that the risk of Mets was linearly increased according to incremental NAFLD severity (from mild to severe). Together, these findings of the current and previous studies suggest that obesity, especially central obesity, increases the odds for NAFL directly and/or indirectly by inducing metabolic disorders, such as metabolic syndrome and IR [37].

In addition to obesity, the findings of the current study are also in accordance with the findings from Korean cohort studies reporting sarcopenia as another novel risk factor of NAFL. In a total of 4398 Korean adults who had no baseline NAFLD, Lee et al. [38] examined the association between ASM and the 7-year follow-up incident of NAFLD, and found that decreased ASM was positively associated with incident of NAFLD independent of obesity. By analyzing a 7-year follow-up study, involving 12,624 Korean adults aged 20 years and older, Kim et al. [14] also showed that baseline skeletal muscle index (SMI) was inversely associated with incident NAFLD, and positively correlated with the resolution of baseline NAFLD. 

The association between sarcopenia and NAFL has been reported from population-based, cross-sectional studies. By analyzing data obtained from KNHANES IV and V, Lee et al. [16] examined the association between sarcopenia and NAFLD in a representative sample of Korean adults, and found that decreased SMI was independently associated with an increased risk of NAFL and NASH in Korean adults. In a recent meta-analysis involving 19 articles, Cai et al. [39] showed that patients with NAFLD had lower SMI than normal controls. In that study, sarcopenic individuals were found to have a significantly higher risk of developing NAFL (OR = 1.33), NASH (OR = 2.42) and hepatic fibrosis (OR = 1.56) when compared with their non-sarcopenic counterparts. Those previous findings emphasize the clinical importance of increases in skeletal muscle mass as a therapeutic strategy against NAFL.

Evidence has been accumulated to support lifestyle intervention as a therapeutic strategy for patients with NAFL. In a review paper, Glass et al. [40] showed that a combination of aerobic and resistance exercise, targeting both CRF and myokines, resulted in the greatest benefits for NAFLD. In a lifestyle intervention with diet modification and increase in physical activity, for example, CRF at baseline was the strongest predictor of reductions in fatty liver, independent of changes in body fatness [41]. Similarly, a 16 weeks of supervised exercise training resulted in improvements in both CRF and NAFL independent of weight loss, and the exercise training-induced improvements in CRF and NAFL were reversed after 1 year of exercise cessation [42], implying that CRF and NAFL are causally related to each other. Consequently, an intervention study will be necessary to elucidate the nature of the link between CRF and NAFL, as well as the underlying mechanism(s).

Several explanations are possible for the high eCRF-related attenuation of the prevalence of NAFL, associated with sarcopenia and central obesity, observed in the current study. First, sarcopenia and obesity are two of the major risk factors that are frequently encountered in patients with NAFL. IR is considered as a common factor linking sarcopenia and obesity to hepatic steatosis, via perturbations in the release of myokines and adipokines, respectively [17]. On the other hand, CRF is positively associated with whole-body insulin sensitivity in apparently healthy men and women [39], as well as overweight/obese subjects [10]. Therefore, it seems reasonable to speculate that high CRF-related insulin sensitivity may attenuate the individual and synergistic impact of sarcopenia and obesity on the risk of hepatic steatosis. 

Second, inflammation may be another explanation for the link between hepatic steatosis, sarcopenia and obesity [43,44]. As such, high CRF-related anti-inflammatory responses may attenuate the individual and synergistic impacts of sarcopenia and obesity on NAFL [11]. 

Third, metabolic syndrome, characterized by hyperglyceridemia, hyperlipidemia and hypertension, may contribute to the development of NAFL [45]. On the other hand, promotion of CRF via regular exercise has been well established as an effective therapeutic strategy for patients with metabolic complications [46,47]. Together, the findings of the current study suggest that sarcopenia and obesity are individually and synergistically associated with whole-body insulin resistance/diabetes, low grade inflammation and metabolic syndrome, contributing to the development of NAFL, while high CRF may alleviate the individual and synergistic impacts of sarcopenia and obesity on NAFL by suppressing/minimizing those metabolic complications.

This study has limitations. First, the cross-sectional nature of this study does not allow causal inference regarding the potential role of eCRF in determining the relationships of sarcopenia and obesity with NAFL. Second, a further study will be necessary to investigate the cellular and molecular mechanism(s) through which CRF alleviates the impact of sarcopenic obesity on the risk of hepatic steatosis. Third, most previous studies involving Korean populations did not include CRF as an exposure, perhaps due to practical the difficulties/limitations related to its measurement. Alternatively, we included eCRF as an important exposure, so that the nature of the relationship of sarcopenic obesity with NAFLD may be better delineated or unveiled [18]. However, the accuracy of the eCRF used in this study remains to be validated in Korean populations. Fourth, BMI and waist-to-hip ratio (WHR) were used as obesity measures, and these are also closely associated with metabolic disorders [46]. Consequently, the impact of the coexistence of sarcopenia and obesity on NAFL should be further addressed in a future study using anthropometric measures of obesity. Fifth, assessment of alcohol consumption based on a self-reported questionnaire may not eliminate the possibility of underestimating the alcohol intake. Lastly, although using HSI and NAFLD-LFS as biomarkers of NAFL was previously validated against ultrasound or ^1^H-MRS [31], their accuracy in quantifying steatosis was also questioned, and contrasted with liver biopsy as the reference standard [48]. Therefore, the accuracy of HSI and NAFLD-LFS should be validated against liver biopsy, ultrasound or ^1^H-MRS as reference standard in a large cohort study using Korean populations.

Despite the limitations, the current findings of this population-based, cross-sectional study indicate the individual and synergistic impacts of sarcopenia and obesity on NAFL, and expand the current knowledge and literature regarding this issue, proving the possible role of eCRF as an important mediator in determining the relationship between sarcopenic obesity and NAFL in Korean adults. 

## 5. Conclusions

This study examined the impacts of sarcopenia and obesity on NAFL in relation to eCRF levels in Korean adults, and found that high eCRF attenuates the individual and synergistic impacts of sarcopenia and obesity on NALF. From a clinical perspective, the findings of our study suggest that the importance of interventions targeting both poor CRF and sarcopenic obesity should be emphasized in the management and prevention of NAFL.

## Figures and Tables

**Table 1:** Description of study participants.

	Women (*n* = 8917)	Men (*n* = 5098)	Total (*n* = 14,015)
**Body fatness and fitness**			
Age (year)	50.3 ± 16.2	49.7 ± 16.4	50.1 ± 16.3
BMI (kg/m^2^)	23.4 ± 3.4	23.9 ± 3.1	23.6 ± 3.3
Body fat (%)	33.0 ± 5.5	22.1 ± 5.5	29.1 ± 7.6
WC (cm)	78.8 ± 9.9	84.0 ± 9.0	80.7 ± 9.9
SMI (%)	26.9 ± 2.7	34.0 ± 3.0	29.5 ± 4.4
RHR (beats/min)	71.6 ± 9.3	70.0 ± 9.4	71.0 ± 9.3
eCRF (METs)	8.2 ± 2.2	11.2 ± 2.2	9.3 ± 2.7
**Socio-economic status**			
Income (10,000 won/month)	345.2 ± 823.7	375.1 ± 1229.8	356.1 ± 990.9
Marital status, *n* (%)			
Married	6308 (70.7)	4055 (79.5)	10,363 (73.9)
Widow/divorced	1742 (19.6)	232 (4.6)	1974 (14.1)
Unmarried	867 (9.7)	811 (15.9)	1678 (12.0)
Education, *n* (%)			
Elementary	3101 (34.8)	923 (18.1)	4024 (28.7)
Middle/high	3672 (41.2)	2426 (47.6)	6098 (43.5)
College	2144 (24.0)	1749 (34.3)	3893 (27.8)
**Health conditions**			
Smoking, *n* (%)	728 (8.2)	3856 (75.6)	4584 (32.7)
Alcohol, *n* (%)	2049 (23.0)	373 (7.3)	2422 (17.3)
Inactivity, *n* (%)	5540 (62.1)	2831 (55.5)	8371 (59.7)
Hypertension, *n* (%)	2602 (29.2)	1939 (38.0)	4541 (32.4)
Diabetes, *n* (%)	766 (9.0)	548 (11.1)	1314 (9.8)
Metabolic syndrome, *n* (%)	2056 (24.2)	1380 (28.0)	3436 (25.6)
Menopause, *n* (%)	4069 (45.6)	0 (0.0)	4069 (29.0)
GLM, *n* (%)	568 (6.4)	381 (7.5)	949 (6.8)
LLM, *n* (%)	499 (5.6)	198 (3.9)	697 (5.0)
**Blood markers**			
FBG (mg/dL)	96.2 ± 21.3	99.7 ± 26.2	97.5 ± 23.3
Insulin (uIU/L)	10.0 ± 5.6	9.9 ± 5.4	10.0 ± 5.5
HOMA-IR	2.4 ± 2.3	2.5 ± 1.7	2.5 ± 2.1
TC (mg/dL)	189.0 ± 36.0	186.7 ± 34.8	188.2 ± 35.6
HDL-C (mg/dL)	49.8 ± 10.9	44.5 ± 10.2	47.8 ± 11.0
TG (mg/dL)	115.0 ± 76.6	147.9 ± 104.6	127.1 ± 89.3
AST (IU/L)	20.3 ± 8.1	23.7 ± 12.2	21.5 ± 10.0
ALT (IU/L)	17.7 ± 12.7	26.2 ± 22.0	20.8 ± 17.2
Platelet (10^9^/L)	264.2 ± 59.7	243.3 ± 54.1	256.5 ± 58.6
**Indices of hepatic steatosis**			
NAFLD-LFS	−1.44 ± 1.44	−1.02 ± 1.47	−1.29 ± 1.47
HSI	32.3 ± 4.8	32.7 ± 5.4	32.4 ± 5.0

BMI, body mass index; WC, waist circumference; SMI, skeletal muscle index; RHR, resting heart rate; eCRF, non-exercise-based estimation of cardiorespiratory fitness; GLM, glucose-lowering medications; LLM, lipid-lowering medications; FBG, fasting blood glucose; HOMA-IR, homeostasis model assessment of insulin resistance; TC, total cholesterol; TG, triglycerides; AST, aspartate aminotransferase; ALT, alanine aminotransferase; NAFLD-LFS, non-alcoholic fatty liver disease-liver fatty score; HSI, hepatic steatosis index.

**Table 2:** The impacts of sarcopenia and obesity on the prevalence of NAFL stratified by eCRF levels.

	Optimal	Sarcopenia	Obesity	Sarcopenic Obesity
Lower eCRF	177 (14.9%)	127 (27.9%)	281 (61.2%)	524 (69.4%)
Middle eCRF	418 (11.2%)	183 (20.7%)	410 (59.8%)	463 (63.3%)
Upper eCRF	217 (9.3%)	68 (18.3%)	121 (49.0%)	94 (54.3%)
	*p* < 0.001	*p* = 0.001	*p* = 0.005	*p* < 0.001

eCRF: non-exercise-based estimation of cardiorespiratory fitness.

**Table 3:** Odds ratio (OR) of sarcopenia and obesity-based phenotypes for NAFL, stratified by eCRF levels (data are presented as OR and 95% confidence interval).

Phenotypes		Lower 25% eCRF		Middle 50% eCRF		Upper 5% eCRF	
SAR	OB	Crude OR	Adjusted OR	Crude OR	Adjusted OR	Crude OR	Adjusted OR
-	-	1 (referent)	1 (referent)	1 (referent)	1 (referent)	1 (referent)	1 (referent)
+	-	2.2 (1.7–2.9) **	2.1 (1.5–3.1) **	2.1 (1.7–2.5) **	1.6 (1.3–2.1) **	2.2 (1.6–3.0) **	2.1 (1.4–3.1) *
-	+	9.0 (7.1–11.6) **	2.9 (2.0–4.2) **	11.8 (9.8–11.4) **	3.5 (2.7–4.6) **	9.4 (7.0–12.5) **	1.8 (1.2–2.8) *
+	+	13.0 (10.4–16.2) **	5.9 (4.3–8.2) **	13.7 (11.4–16.4) **	4.2 (3.2–5.5) **	11.6 (8.3–16.1) **	2.5 (1.5–4.1) **

eCRF, non-exercise-based estimation of cardiorespiratory fitness; SAR, sarcopenia; OB, obesity. Minus (-) and plus (+) indicate the absence of SAR or OB and the presence of SAR and/or OB, respectively. Adjusted for income, marital status, education, resting heart rate, smoking, alcohol consumption, hypertension and menopause. * *p* < 0.05, ** *p* < 0.001.

## Supplementary Materials

The following are available online at https://www.mdpi.com/1660-4601/17/11/3902/s1, Table S1: Descriptive statistics of sarcopenic obesity-based phenotypes in lower eCRF category, Table S2: Descriptive statistics of sarcopenic obesity-based phenotypes in middle eCRF category, Table S3: Descriptive statistics of sarcopenic obesity-based phenotypes in upper eCRF category.

## Abbreviations

CRF	cardiorespiratory fitness
eCRF	estimated cardiorespiratory fitness
WC	waist circumference
WHR	waist-to-hip ratio
BMI	body mass index
OR	odds ratio
CI	confidence interval
NAFLD	non-alcoholic fatty liver disease
NAFL	non-alcoholic fatty liver disease
NASH	non-alcoholic
CVDs	cardiovascular diseases
KNHNES	Korea National Health and Nutrition Examination Surveys
ASM	Appendic ular skeletal muscle mass
SMI	skeletal muscle index
DEXA	dual energy X-ray absorptiometry
METs	metabolic equivalents
NAFLD-LFS	NAFLD liver fat score
HSI	hepatic steatosis index
Mets	metabolic syndrome
RHR	resting heart rate
SBP	systolic blood pressure
DBP	diastolic blood pressure
TC	total cholesterol
TG	triglycerides
HDLC	high-density lipoprotein cholesterol
FBG	fasting blood glucose
ALT	alanine aminotransferase
AST	aspartate aminotransferase
HOMA-IR	homeostasis model assessment of insulin resistance
GLM	glucose-lowering medications
LLM	lipid-lowering medications

## References

[1] Araújo A.R., Rosso N., Bedogni G., Tiribelli C., Bellentani S. (2018). Global epidemiology of non-alcoholic fatty liver disease/non-alcoholic steatohepatitis: What we need in the future. Liver Int..

[2] Sherif Z.A., Saeed A., Ghavimi S., Nouraie S.M., Laiyemo A.O., Brim H., Ashktorab H. (2016). Global epidemiology of nonalcoholic fatty liver disease and prespectives on US minority populations. Dig. Dis. Sci..

[3] Fan J.G., Kim S.U., Wong V.W. (2017). New trends on obesity and NAFLD in Asia. J. Hepatol..

[4] Younossi Z.M. (2019). Non-alcoholic fatty liver disease—A global public health perspective. J. Hepatol..

[5] Ballestri S., Zona S., Targher G., Romagnoli D., Baldelli E., Nascimbeni F., Roverato A., Guaraldi G., Lonardo A. (2016). Nonalcoholic fatty liver disease is associated with an almost twofold increased risk of incident type 2 diabetes and metabolic syndrome. Evidence from a systematic review and meta-analysis. J. Gastroenterol. Hepatol..

[6] Targher G., Byrne C.D., Lonardo A., Zoppini G., Barbui C. (2016). Non-alcoholic fatty liver disease and risk of incident cardiovascular disease: A meta-analysis. J. Hepatol..

[7] Do K., Brown R.E., Wharton S., Ardern C.I., Kuk J.L. (2018). Association between cardiorespiratory fitness and metabolic risk factors in a population with mild to severe obesity. BMC Obes..

[8] Lee J., Cho Y.K., Kang Y.M., Kim H.S., Jung C.H., Kim H.K., Park J.Y., Lee W.J. (2019). The impact of NAFLD and waist circumference changes on diabetes development in prediabetes subjects. Sci. Rep..

[9] Chen L.Y., Zmora R., Duval S., Chow L.S., Lloyd-Jones D.M., Schreiner P.J. (2019). Cardiorespiratory fitness, adiposity, and heart rate variability: The coronary artery risk development in young adults study. Med. Sci. Sports Exerc..

[10] Haufe S., Engeli S., Budziarek P., Utz W., Schulz-Menger J., Hermsdorf M., Wiesner S., Otto C., Haas V., de Greiff A. (2010). Cardiorespiratory fitness and insulin sensitivity in overweight or obese subjects may be linked through intrahepatic lipid content. Diabetes.

[11] Madssen E., Skaug E.A., Wisløff U., Ellingsen Ø., Videm V. (2019). Inflammation is strongly associated with cardiorespiratory fitness, sex, BMI, and the metabolic syndrome in a self-reported healthy population: HUNT3 fitness study. Mayo Clin. Proc..

[12] Wijarnpreecha K., Kim D., Raymond P., Scribani M., Ahmed A. (2019). Associations between sarcopenia and nonalcoholic fatty liver disease and advanced fibrosis in the USA. Eur. J. Gastroenterol. Hepatol..

[13] Peng T.C., Wu L.W., Chen W.L., Liaw F.Y., Chang Y.W., Kao T.W. (2019). Nonalcoholic fatty liver disease and sarcopenia in a Western population (NHANES III): The importance of sarcopenia definition. Clin. Nutr..

[14] Kim G., Lee S.E., Lee Y.B., Jun J.E., Ahn J., Bae J.C., Jin S.M., Hur K.Y., Jee J.H., Lee M.K. (2018). Relationship between relative skeletal muscle mass and non-alcoholic fatty liver disease: A 7-year longitudinal study. Hepatology.

[15] Koo B., Kim D., Joo S., Kim J., Chang M., Kim B., Lee K., Kim W. (2017). Sarcopenia is an independent risk factor for non-alcoholic steatohepatitis and significant fibrosis. J. Hepatol..

[16] Lee Y.H., Kim S.U., Song K., Park J.Y., Kim D.Y., Ahn S.H., Lee B.W., Kang E.S., Cha B.S., Han K.H. (2016). Sarcopenia is associated with significant liver fibrosis independently of obesity and insulin resistance in nonalcoholic fatty liver disease: Nationwide surveys (KNHANES 2008-2011). Hepatology.

[17] Merli M., Lattanzi B., Aprile F. (2019). Sarcopenic obesity in fatty liver. Curr. Opin. Clin. Nutr. Metab. Care.

[18] Jeong E.H., Jun D.W., Cho Y.K., Choe Y.G., Ryu S., Lee S.M., Jang E.C. (2013). Regional prevalence of non-alcoholic fatty liver disease in Seoul and Gyeonggi-do, Korea. Clin. Mol. Hepatol..

[19] Kim T.N., Park M.S., Kim Y.J., Lee E.J., Kim M.K., Kim J.M., Ko K.S., Rhee B.D., Won J.C. (2014). Association of low muscle mass and combined low muscle mass and visceral obesity with low cardiorespiratory fitness. PLoS ONE.

[20] Jurca R., Jackson A.S., LaMonte M.J., Morrow J.R., Blair S.N., Wareham N.J., Haskell W.L., van Mechelen W., Church T.S., Jakicic J.M. (2005). Assessing cardiorespiratory fitness without performing exercise testing. Am. J. Prev. Med..

[21] Korean Centers for Disease Control and Prevention Korea National Health and Nutrition Examination Surveys. http://knhanes.cdc.go.kr.

[22] Kweon A., Kim Y., Jang M.J., Kim Y., Kim K., Choi S., Chun C., Khang Y.H., Oh K. (2014). Data resource profile: The Korea National Health and Nutrition Examination Survey (KNHANES). Int. J. Epidemiol..

[23] Mirsa A. (2015). Ethnic-specific criteria for classification of body mass index: A perspective for Asian Indians and American Diabetes Association Position Statement. Diabetes Technol. Ther..

[24] Sayer A.A., Syddall H., Martin H., Patel H., Baylis D., Cooper C. (2008). The developmental origins of sarcopenia. J. Nutr. Health Aging.

[25] Fox C.S., Massaro J.M., Hoffmann U., Pou K.M., Maurovich-Horvat P., Liu C.Y., Vasan R.S., Murabito J.M., Meigs J.B., Cupples L.A. (2007). Abdominal visceral and subcutaneous adipose tissue compartments: Association with metabolic risk factors in the Framingham Heart Study. Circulation.

[26] Pouliot M.C., Després J.P., Lemieux S., Moorjani S., Bouchard C., Tremblay A., Nadeau A., Lupien P.J. (1994). Waist circumference and abdominal sagittal diameter: Best simple anthropometric indexes of abdominal visceral adipose tissue accumulation and related cardiovascular risk in men and women. Am. J. Cardiol..

[27] Cho G.J., Yoo H.J., Hwang S.Y., Choi J., Lee K.M., Choi K.M., Baik S.H., Han S.W., Kim T. (2018). Differential relationship between waist circumference and mortality according to age, sex, and body mass index in Korean with age of 30–90 years; a nationwide health insurance database study. BMC Med..

[28] Lee S.Y., Park H.S., Kim D.J., Han J.H., Kim S.M., Cho G.J., Kim D.Y., Kwon H.S., Kim S.R., Lee C.B. (2007). Appropriate waist circumference cutoff points for central obesity in Korean adults. Diabetes Res. Clin. Pract..

[29] Lee Y.H., Jung K.S., Kim S.U., Yoon H.J., Yun Y.J., Lee B.W., Kang E.S., Han K.H., Lee H.C., Cha B.S. (2015). Sarcopaenia is associated with NAFLD independently of obesity and insulin resistance: Nationwide surveys (KNHANES 2008-2011). J. Hepatol..

[30] Ross R.M., Jackson A.S. (1990). Exercise Concepts, Calculations, and Computer Applications.

[31] Kotronen A., Peltonen M., Hakkarainen A., Sevastianova K., Bergholm R., Johansson L.M., Lundbom N., Rissanen A., Ridderstråle M., Groop L. (2009). Prediction of non-alcoholic fatty liver disease and liver fat using metabolic and genetic factors. Gastroenterology.

[32] Grundy S.M., Brewer H.B., Cleeman J.I., Smith S.C., Lenfant C. (2004). National Heart, Lung, and Blood Institute; American Heart Association. Definition of metabolic syndrome: Report of the National Heart, Lung, and Blood Institute/American Heart Association conference on scientific issues related to definition. Arterioscler. Thromb. Vasc. Biol..

[33] Lee J.H., Kim D., Kim H.J., Lee C.H., Yang J.I., Kim W., Kim Y.J., Yoon J.H., Cho S.H., Sung M.W. (2010). Hepatic steatosis index: A simple screening tool reflecting nonalcoholic fatty liver disease. Dig. Liver Dis..

[34] Chang Y., Jung H.S., Cho J., Zhang Y., Yun K.E., Lazo M., Pastor-Barriuso R., Ahn J., Kim C.W., Rampal S. (2016). Metabolically healthy obesity and the development of nonalcoholic fatty liver disease. Am. J. Gastroenterol..

[35] Kwon Y.M., Oh S.W., Hwang S.S., Lee C., Kwon H., Chung G.E. (2012). Association of non-alcoholic fatty liver disease with components of metabolic syndrome according to body mass index in Korean adults. Am. J. Gastroenterol..

[36] Yang K.C., Hung H.F., Lu C.W., Chang H.H., Lee L.T., Huang K.C. (2016). Association of non-alcoholic fatty liver disease with metabolic syndrome independently of central obesity and insulin resistance. Sci. Rep..

[37] Polyzos S.A., Kountouras J., Mantzoros C.S. (2019). Obesity and nonalcoholic fatty liver disease: From pathophysiology to therapeutics. Metabolism.

[38] Lee M.J., Kim E.H., Bae S.J., Kim G.A., Park S.W., Choe J., Jung C.H., Lee W.J., Kim H.K. (2019). Age-related decrease in skeletal muscle mass is an independent risk factor for incident nonalcoholic fatty liver disease: A 10-year retrospective cohort study. Gut Liver.

[39] Cai C., Song X., Chen Y., Chen X., Yu C. (2020). Relationship between relative skeletal muscle mass and nonalcoholic fatty liver disease: A systematic review and meta-analysis. Hepatol. Int..

[40] Glass O.K., Radia A., Kraus W.E., Abdelmalek M.F. (2017). Exercise Training as Treatment of Nonalcoholic Fatty Liver Disease. J. Funct. Morphol. Kinesiol..

[41] Kantartzis K., Thamer C., Peter A., Machann J., Schick F., Schraml C., Konigsrainer A., Konigsrainer I., Krober S., Niess A. (2009). High cardiorespiratory fitness is an independent predictor of the reduction in liver fat during a lifestyle intervention in non-alcoholic fatty liver disease. Gut.

[42] Pugh C.J., Sprung V.S., Jones H., Richardson P., Shojaee-Moradie F., Umpleby A.M., Green D.J., Cable N.T., Trenell M.I., Kemp G.J. (2016). Exercise-induced improvements in liver fat and endothelial function are not sustained 12 months following cessation of exercise supervision in nonalcoholic fatty liver disease. Int. J. Obes..

[43] Clarke S.L., Reaven G.M., Leonard D., Barlow C.E., Haskell W.L., Willis B.L., DeFina L., Knowles J.W., Maron D.J. (2020). Cardiorespiratory fitness, body mass index, and markers of insulin resistance in apparently health women and men. Am. J. Med..

[44] Henningsen J., Rigbolt K.T., Blagoev B., Pedersen B.K., Kratchmarova I. (2010). Dynamics of the skeletal muscle secretome during myoblast differentiation. Mol. Cell Proteomics.

[45] Wree A., Kahraman A., Gerken G., Canbay A. (2011). Obesity affects the liver—The link between adipocytes and hepatocytes. Digestion.

[46] Hadi H.E., Vincenzo A.D., Vettor R., Rossato M. (2019). Cardio-metabolic disorders in non-alcoholic fatty liver disease. Int. J. Mol. Sci..

[47] Polyzos S.A., Margioris A.N. (2018). Sarcopeni obesity. Hormones.

[48] Fedchuk L., Nascimbeni F., Pais R., Charlotte F., Housset C., Ratziu V., LIDO Study Group (2014). Performance and limitations of steatosis biomarkers in patients with nonalcoholic fatty liver disease. Aliment. Pharmacol. Ther..

