# Supplementary material for: Estimated Cardiorespiratory Fitness Attenuates the Impacts of Sarcopenia and Obesity on Non-Alcoholic Fatty Liver in Korean Adults
Source: Int J Environ Res Public Health. 2020 May 31;17(11):3902. doi: 10.3390/ijerph17113902 (PMC7312192; doi:10.3390/ijerph17113902)
Supplement: Supplementary file 1 [file ijerph-17-03902-s001.pdf]

Supplementary Materials

Table 1. Descriptive statistics of sarcopenic obesity-based phenotypes in lower eCRF category.

Table 2. Descriptive statistics of sarcopenic obesity-based phenotypes in middle eCRF category.

Table 3. Descriptive statistics of sarcopenic obesity-based phenotypes in upper eCRF category.

Table 1. Descriptive statistics of sarcopenic obesity-based phenotypes in lower eCRF category

	Sarcopenia (-)/obesity (-) (n = 1,408)	Sarcopenia (-)/obesity (+) (n = 565)	Sarcopenia (+)/obesity (-) (n = 569)	Sarcopenia (+)/obesity (+) (n = 959)	*p* for trends
**Body fatness and fitness**					
Women, n (%)	866 (61.5)	346 (61.2)	374 (65.7)	640 (66.7)	0.004
Age (year)	48.2±18.5	51.8±16.2	54.5±18.2	58.8±16.5	<0.001
BMI (kg/m^2)	23.0±2.3	27.1±2.5	24.3±2.1	28.2±3.2	<0.001
Body fat (%)	27.4±6.4	30.4±5.8	34.2±5.9	36.4±6.0	<0.001
WC (cm)	78.1±6.5	92.6±4.9	80.8±5.0	94.7±6.7	<0.001
SMI (%)	30.5±3.7	29.6±3.5	26.4±3.2	25.6±3.4	<0.001
RHR (beats/min)	75.3±9.8	72.4±9.9	74.6±10.0	73.7±9.8	0.058
eCRF (METs)	8.4±2.2	7.5±2.3	7.5±2.1	6.4±2.3	<0.001
**Socio-economic status**					
Income (10,000 won/month)	353.2±766.1	300.9±505.3	386.5±930.8	269.2±347.0	0.075
Marital status, n (%)					0.001
Married	973 (69.1)	422 (74.7)	412 (72.4)	648 (67.6)	
Widow/divorced	215 (15.3)	99 (17.5)	112 (19.7)	258 (26.9)	
Unmarried	220 (15.6)	44 (7.8)	45 (7.9)	53 (5.5)	
Education, n (%)					<0.001
Elementary	357 (25.4)	201 (35.6)	186 (32.7)	470 (49.0)	
Middle/high	604 (42.9)	235 (41.6)	240 (42.2)	334 (34.8)	
College	447 (31.7)	129 (22.8)	143 (25.1)	155 (16.2)	
**Health conditions**					
Smoking, n (%)	494 (35.1)	189 (33.5)	190 (33.4)	298 (31.1)	0.049
Alcohol, n (%)	226 (16.1)	105 (18.6)	103 (18.1)	234 (24.4)	<0.001
Inactivity, n (%)	1,393 (98.9)	538 (95.2)	564 (99.1)	907 (94.6)	<0.001
Hypertension, n (%)	393 (27.9)	249 (44.1)	217 (38.1)	486 (50.7)	<0.001
Diabetes, n (%)	90 (6.8)	80 (14.9)	72 (13.9)	183 (21.1)	<0.001
Metabolic syndrome, n (%)	167 (12.5)	357 (66.4)	109 (21.0)	595 (68.5)	<0.001
Menopause, n (%)	279 (19.8)	180 (31.9)	168 (29.5)	433 (45.2)	<0.001
GLM, n (%)	57 (4.0)	46 (8.1)	57 (10.0)	154 (16.1)	<0.001
LLM, n (%)	42 (3.0)	40 (7.1)	29 (5.1)	90 (9.4)	<0.001
**Blood markers**					
FBG (mg/dL)	96.0±22.6	102.6±24.9	100.8±27.2	108.1±30.4	<0.001
Insulin (uIU/L)	9.9±6.0	12.8±6.2	11.4±11.5	13.7±7.3	<0.001
HOMA-IR	2.4±1.7	3.3±2.0	3.0±5.6	3.7±2.6	<0.001
TC (mg/dL)	184.5±34.9	194.9±36.1	195.2±37.0	198.9±38.3	<0.001
HDL-C (mg/dL)	47.6±10.6	42.8±9.0	46.7±10.3	43.8±9.7	<0.001
TG (mg/dL)	124.4±95.8	164.3±109.2	130.1±69.6	163.9±98.5	<0.001
AST (IU/L)	20.1±6.9	23.0±8.1	21.7±8.3	24.7±12.0	<0.001
ALT (IU/L)	19.0±11.8	26.2±16.1	22.2±15.9	28.3±23.1	<0.001
Platelet (10^9/L)	259.9±58.2	259.0±59.4	267.9±65.5	261.9±64.7	0.091
**Indices of hepatic steatosis**					
NAFLD-LFS	-1.54±1.34	-0.11±1.53	-1.00±2.09	0.17±1.67	<0.001
HSI	31.8±3.9	37.4±4.4	33.9±3.9	38.8±5.1	<0.001

BMI, body mass index; WC, waist circumference; SMI, skeletal muscle index; RHR, resting heart rate; eCRF, non-exercise-based estimation of cardiorespiratory fitness; GLM, glucose-lowering medications; LLM, lipid-lowering medications; FBG, fasting blood glucose; HOMA-IR, homeostasis model assessment of insulin resistance; TC, total cholesterol; TG, triglycerides; AST, asparate transaminase; ALT, alanine aminotransferase; non-alcoholic fatty liver disease-liver fatty score; HSI, hepatic steatosis index.

Table 2. Descriptive statistics of sarcopenic obesity-based phenotypes in middle eCRF category

Measured parameters	Sarcopenia (-)/obesity (-) (n = 4,325)	Sarcopenia (-)/obesity (+) (n = 781)	Sarcopenia (+)/obesity (-) (n = 1,044)	Sarcopenia (+)/obesity (+) (n = 870)	*p* for trends
**Body fatness and fitness**					
Women, n (%)	2,707 (62.6)	444 (56.9)	727 (69.6)	589 (67.7)	<0.001
Age (year)	46.3±15.8	53.1±13.7	53.1±15.1	57.6±13.0	<0.001
BMI (kg/m^2)	21.7±2.4	26.5±2.3	23.4±2.3	27.4±2.7	<0.001
Body fat (%)	26.0±6.6	29.6±5.9	34.1±5.7	35.9±5.8	<0.001
WC (cm)	75.2±7.3	91.7±5.2	79.1±6.2	93.3±5.9	<0.001
SMI (%)	31.1±4.0	30.1±3.6	26.2±3.1	25.9±3.3	<0.001
RHR (beats/min)	70.5±8.9	70.9±9.0	70.3±9.2	70.8±9.0	0.815
eCRF (METs)	9.6±2.3	9.1±2.6	8.5±2.2	8.1±2.4	<0.001
**Socio-economic status**					
Income (10,000 won/month)	365.5±607.8	310.2±534.9	432.1±2266.1	336.6±1002.6	0.786
Marital status, n (%)					<0.001
Married	3,188 (73.7)	634 (81.2)	786 (75.3)	646 (74.3)	
Widow/divorced	449 (10.4)	105 (13.4)	167 (16.0)	185 (21.2)	
Unmarried	688 (15.9)	42 (5.4)	91 (8.7)	39 (4.5)	
Education, n (%)					<0.001
Elementary	916 (21.2)	272 (34.8)	325 (31.1)	412 (47.4)	
Middle/high	1,939 (44.8)	347 (44.4)	425 (40.7)	325 (37.3)	
College	1,470 (34.0)	162 (20.8)	294 (28.2)	133 (15.3)	
**Health conditions**					
Smoking, n (%)	1,415 (32.7)	295 (37.8)	293 (28.1)	271 (31.1)	0.061
Alcohol, n (%)	610 (14.1)	139 (17.8)	216 (20.7)	216 (24.8)	<0.001
Inactivity, n (%)	3,229 (74.7)	329 (42.1)	748 (71.6)	385 (44.3)	<0.001
Hypertension, n (%)	1,060 (24.5)	336 (43.0)	386 (37.0)	432 (49.7)	<0.001
Diabetes, n (%)	242 (5.8)	146 (19.0)	110 (11.1)	159 (19.2)	<0.001
Metabolic syndrome, n (%)	417 (10.0)	480 (62.6)	186 (18.7)	561 (67.6)	<0.001
Menopause, n (%)	924 (21.4)	275 (35.2)	396 (37.9)	432 (49.7)	<0.001
GLM, n (%)	170 (3.9)	101 (12.9)	83 (8.0)	119 (13.7)	<0.001
LLM, n (%)	128 (3.0)	60 (7.7)	53 (5.1)	114 (13.1)	<0.001
**Blood markers**					
FBG (mg/dL)	93.8±20.0	105.1±28.7	97.8±24.1	105.5±28.6	<0.001
Insulin (uIU/L)	8.8±3.4	11.7±5.4	9.8±6.2	12.8±6.8	<0.001
HOMA-IR	2.0±1.0	3.1±1.9	2.5±3.6	3.4±2.3	<0.001

TC (mg/dL)	182.5±33.7	195.2±34.6	193.1±35.9	197.8±36.9	<0.001
HDL-C (mg/dL)	49.6±11.2	43.6±9.6	47.2±10.4	44.3±9.8	<0.001
TG (mg/dL)	111.4±83.2	158.2±92.2	133.6±82.2	164.0±97.1	<0.001
AST (IU/L)	20.4±8.1	24.5±12.5	20.8±7.1	24.1±10.6	<0.001
ALT (IU/L)	18.3±13.6	27.8±21.5	19.5±12.7	26.2±18.8	<0.001
Platelet (10^9/L)	253.9±56.9	253.8±60.4	261.9±59.5	261.5±58.3	<0.001
**Indices of hepatic steatosis**					
NAFLD-LFS	-1.80±0.99	-0.20±1.46	-1.41±1.43	-0.05±1.58	<0.001
HSI	30.0±3.8	36.7±4.1	32.3±3.8	37.5±4.3	<0.001

BMI, body mass index; WC, waist circumference; SMI, skeletal muscle index; RHR, resting heart rate; eCRF, non-exercise-based estimation of cardiorespiratory fitness; GLM, glucose-lowering medications; LLM, lipid-lowering medications; FBG, fasting blood glucose; HOMA-IR, homeostasis model assessment of insulin resistance; TC, total cholesterol; TG, triglycerides; AST, asparate transaminase; ALT, alanine aminotransferase; non-alcoholic fatty liver disease-liver fatty score; HSI, hepatic steatosis index.

Table 3. Descriptive statistics of sarcopenic obesity-based phenotypes in upper eCRF category

Measured parameters	Sarcopenia (-)/obesity (-) (n = 2,589)	Sarcopenia (-)/obesity (+) (n = 284)	Sarcopenia (+)/obesity (-) (n = 423)	Sarcopenia (+)/obesity (+) (n = 198)	*p* for trends
**Body fatness and fitness**					
Women, n (%)	1,559 (60.2)	202 (71.1)	312 (73.8)	151 (76.3)	<0.001
Age (year)	46.4±15.1	56.6±12.2	51.9±14.1	58.9±11.4	<0.001
BMI (kg/m^2)	21.8±2.3	25.5±1.7	23.3±2.2	26.5±1.8	<0.001
Body fat (%)	24.9±7.0	30.3±5.7	34.6±5.7	36.3±5.4	<0.001
WC (cm)	75.0±7.0	89.8±3.7	78.4±6.1	91.1±4.1	<0.001
SMI (%)	31.7±4.3	29.2±3.3	26.0±3.0	25.4±3.0	<0.001
RHR (beats/min)	68.7±8.7	68.0±7.9	68.4±8.6	68.1±8.3	0.513
eCRF (METs)	11.7±2.1	9.8±2.0	10.5±1.9	9.3±1.9	<0.001
**Socio-economic status**					
Income (10,000 won/month)	391.6±1250.5	255.2±232.2	326.7±253.1	270.4±297.7	0.250
Marital status, n (%)					0.005
Married	1,963 (75.8)	220 (77.5)	322 (76.2)	149 (75.3)	
Widow/divorced	232 (9.0)	52 (18.3)	59 (13.9)	41 (20.7)	
Unmarried	394 (15.2)	12 (4.2)	42 (9.9)	8 (4.0)	
Education, n (%)					<0.001
Elementary	536 (20.7)	136 (47.9)	111 (26.2)	102 (51.5)	
Middle/high	1,258 (48.6)	115 (40.5)	207 (48.9)	69 (34.8)	
College	795 (30.7)	33 (11.6)	105 (24.9)	27 (13.7)	
**Health conditions**					
Smoking, n (%)	907 (35.0)	79 (27.8)	108 (25.5)	45 (22.7)	<0.001
Alcohol, n (%)	387 (14.9)	56 (19.7)	81 (19.1)	49 (24.7)	<0.001
Inactivity, n (%)	229 (8.8)	15 (5.3)	27 (6.4)	7 (3.5)	0.001
Hypertension, n (%)	603 (23.3)	121 (42.6)	158 (37.4)	100 (50.5)	<0.001
Diabetes, n (%)	110 (4.3)	48 (17.2)	33 (8.0)	41 (20.9)	<0.001
Metabolic syndrome, n (%)	203 (8.0)	173 (62.0)	62 (15.0)	126 (64.3)	<0.001
Menopause, n (%)	561 (21.7)	138 (48.6)	167 (39.5)	116 (58.6)	<0.001
GLM, n (%)	69 (2.7)	35 (12.3)	25 (5.9)	33 (16.7)	<0.001
LLM, n (%)	68 (2.6)	18 (6.3)	34 (8.0)	21 (10.6)	<0.001
**Blood markers**					
FBG (mg/dL)	92.8±16.6	102.7±24.1	97.1±21.6	105.8±26.2	<0.001
Insulin (uIU/L)	8.5±3.5	10.2±4.0	9.6±4.1	11.3±4.9	<0.001
HOMA-IR	2.0±1.0	2.6±1.5	2.3±1.3	3.0±1.6	<0.001

TC (mg/dL)	182.6±33.9	197.7±35.8	193.9±36.0	201.7±39.3	<0.001
HDL-C (mg/dL)	50.6±11.2	45.0±10.2	48.8±11.3	46.4±10.5	0.001
TG (mg/dL)	105.0±76.4	148.7±99.4	127.0±73.8	150.4±93.0	<0.001
AST (IU/L)	21.4±13.5	21.9±7.6	20.6±6.7	22.9±7.7	0.227
ALT (IU/L)	18.7±22.0	21.7±10.8	18.5±10.4	22.6±11.0	0.059
Platelet (10^9/L)	251.2±56.0	256.2±57.6	254.9±59.1	263.2±57.7	0.009
**Indices of hepatic steatosis**					
NAFLD-LFS	-1.85±1.03	-0.62±1.24	-1.55±1.10	-0.39±1.27	<0.001
HSI	29.8±3.6	35.1±3.2	32.0±3.4	36.3±3.3	<0.001

BMI, body mass index; WC, waist circumference; SMI, skeletal muscle index; RHR, resting heart rate; eCRF, non-exercise-based estimation of cardiorespiratory fitness; GLM, glucose-lowering medications; LLM, lipid-lowering medications; FBG, fasting blood glucose; HOMA-IR, homeostasis model assessment of insulin resistance; TC, total cholesterol; TG, triglycerides; AST, asparate transaminase; ALT, alanine aminotransferase; non-alcoholic fatty liver disease-liver fatty score; HSI, hepatic steatosis index.
